# Supplementary material for: Survival in People Living with HIV with or without Recurrence of Hepatocellular Carcinoma after Invasive Therapy
Source: Cancers (Basel). 2023 Mar 8;15(6):1653. doi: 10.3390/cancers15061653 (PMC10046370; doi:10.3390/cancers15061653)
Supplement: Supplementary file 1 [file cancers-15-01653-s001.zip › Table S1 supplemental recurrence HCC.pdf]

Table S1 Supplemental. Characteristics at baseline evaluation (HCC diagnosis) of PLWH who received invasive therapy according to the outcome recurrence.

Variable		Overall (number=41)	RE (number=19)	No-RE (number=22)	P-value
Age (years)		53 (49 - 56)	54.6 (49.5 - 57.5)	52 (47.7 - 56)	0.314
Sex, male		34 (82.9)	13 (68.4)	21 (95.5)	0.036
Years of HIV infection		22.7 (12.7 - 26.4)	24.7 (10.9 - 28.3)	21.3 (12.7 - 25.3)	0.327
Years since first ART		11.9 (7.3 - 16.9)	10.7 (3.72 - 17.91)	12.5 (8.8 - 16.7)	0.745
HIV risk factor					0.658
	Heterosexual	4 (9.8)	3 (15.8)	1 (4.5)	
	IVDU	22 (53.7)	9 (47.4)	13 (59.1)	
	MSM	4 (9.8)	2 (10.5)	2 (9.1)	
	Unknown	11 (26.8)	5 (26.3)	6 (27.3)	
Time to FU, years		4 (1.1 - 7)	4.5 (1.4 - 8.6)	2.7 (0.8 - 6.3)	0.302
N. of nodules >3		3 (2 - 4)	2.6 (2 - 3.5)	3 (2.3 - 4)	0.487
Cancer embolus in portal vein	No	29 (70.7)	14 (73.7)	15 (68.2)	1.000
	Yes	12 (29.3)	5 (26.3)	7 (31.8)	
Extra-hepatic	No	37 (90.2)	17 (89.5)	20 (90.9)	0.729
	Yes	4 (9.8)	2 (10.5)	2 (9.1)	
AFP, median, ng/mL		27.8 (8.7 - 135.2)	29.6 (18.3 - 256)	14.85 (8.5 - 99.9)	0.293
AFP, ng/mL					0.330
	<28.8	19 (51.4)	7 (41.2)	12 (60)	
	≥28.8	18 (48.6)	10 (58.8)	8 (40)	
AST, U/L		70 (34 - 100)	43 (33 - 100)	70.5 (36 - 103.5)	0.64
ALT, U/L		67 (33 - 99)	54 (26 - 93)	68 (40.5 - 99.5)	0.358
Bilirubin, mg/dL		1 (0.78 - 1.75)	0.97 (0.78 - 1.75)	1.02 (0.83 - 1.59)	0.903
PCHE, KU/L		5.07 (3.03 - 6.66)	4.13 (3.03 - 7.95)	5.3 (2.14 - 6.61)	0.896
Albumin, g/L		39.9 (37.26 - 41.11)	40.92 (37.26 - 42.89)	39.11 (33.78 - 40.88)	0.353
CD4 cells count, mmc		433 (262 - 722)	539 (341 - 785)	345 (262 - 614)	0.369
CD8 cells count, mmmc		732 (445 - 1209)	679 (415.5 - 1147)	757 (449 - 1209)	0.935
CD4/CD8 ratio		0.68 (0.34 - 0.98)	0.74 (0.42 - 1.07)	0.51 (0.31 - 0.98)	0.28
Neutrophils, 10^9/L		2.85 (2.4 - 3.4)	2.8 (1.85 - 3.35)	2.85 (2.4 - 3.5)	0.492
Lymphocytes, 10^9/L		1.7 (1.2 - 2.6)	1.85 (1.1 - 2.6)	1.6 (1.4 - 2.4)	0.985
Neutrophils/ lymphocytes ratio		1.58 (1.12 - 2.14)	1.36 (1.06 - 1.85)	1.87 (1.23 - 2.36)	0.285

Variable		Overall (number=41)	RE (number=19)	No-RE (number=22)	P-value
Platelets, 10^9/L		107 (64 - 141)	102 (63 - 124)	108.5 (75 - 152)	0.528
PTs/INR		1.06 (1.03 - 1.28)	1.06 (1.06 - 1.28)	1.08 (0.99 - 1.14)	0.721
Creatinine, mg/dL		0.74 (0.68 - 0.86)	0.81 (0.68 - 1.03)	0.73 (0.68 - 0.79)	0.279
Anti-HCV positive		32 (78)	13 (68.4)	19 (86.4)	0.332
HBsAg positive		8 (19.5)	5 (26.3)	3 (13.6)	0.562
HIV-RNA, copies/mL		2363 (1079 - 14948)	2363 (1900 - 2825)	13665 (258 - 27071)	0.999
HIV-RNA, ≥50copies/mL		4 (9.8)	2 (10.5)	2 (9.1)	0.917
	Unknown	14 (34.1)	7 (36.8)	7 (31.8)	

Results are described by median (IQR) or frequency (%). Abbreviations: n: number; ART: antiretroviral therapy; IVDU: intravenous drug users; MSM: a man who have sex with a man; FU: follow up; AFP: alpha phetoprotein; PTs/INR: prothrombin, second/international normalized ratio; AST: aspartate aminotransferase (normal values <35 IU/L); ALT: alanine aminotransferase (normal values <59 IU/L); PCHE: pseudocholinesterase; HCV: hepatitis C virus; HBsAg: hepatitis B surface antigen.
