# Supplementary material for: Survival in People Living with HIV with or without Recurrence of Hepatocellular Carcinoma after Invasive Therapy
Source: Cancers (Basel). 2023 Mar 8;15(6):1653. doi: 10.3390/cancers15061653 (PMC10046370; doi:10.3390/cancers15061653)
Supplement: Supplementary file 1 [file cancers-15-01653-s001.zip › Table S2 supplemental recurrence HCC.pdf]

Table S2 Supplemental. Characteristics of PLWH at invasive therapy according to the outcome recurrence

**Variable**		**Overall (number=41)**	**RE (number=19)**	**No-RE (number=22)**	**P-value**
First therapy					0.171
	OLT	9 (22.0)	2 (10.5)	7 (31.8)	
	LR	6 (14.6)	4 (21.1)	2 (9.1)	
	RFTA	7 (17.1)	5 (26.3)	2 (9.1)	
	CRE	19 (46.3)	8 (42.1)	11 (50.0)	
Days to therapy from diagnosis		57 (27 - 218)	35 (15 - 122)	109 (31 - 327)	0.097
Years to recurrence from diagnosis		1.34 (0.56 - 3.04)	1.34 (0.56 - 3.04)	. (. - .)	<0.0001
Days to death from recurrence		336 (233.5 - 566.5)	336 (233.5 - 566.5)	. (. - .)	<0.0001
AFP, ng/mL		22.6 (7.7 - 216.2)	78.6 (16.15 - 370)	11.1 (5.5 - 46.3)	0.101
AFP, ng/mL					0.182
	<28.8	20 (57.1)	7 (43.8)	13 (68.4)	
	≥28.8	15 (42.9)	9 (56.3)	6 (31.6)	
AST, U/L		58.5 (32.5 - 130.5)	52.5 (36 - 83)	58.5 (28 - 176)	0.625
ALT, U/L		43 (28 - 96)	45 (29 - 83)	39 (28 - 96)	0.832
Bilirubin, mg/dL		0.9 (0.57 - 2.07)	0.9 (0.6 - 1.64)	0.98 (0.51 - 2.3)	0.807
PCHE, KU/L		6.17 (3.75 - 7.91)	6.2 (4.19 - 6.79)	5.98 (3.75 - 8.12)	0.592
CD4 cells count, mmc		368 (249.5 - 561)	420 (231 - 610)	350 (282 - 512)	0.838
CD8 cells count, mmc		552 (379.5 - 915)	412 (311 - 716)	669.5 (385 - 942)	0.206
CD4/CD8 ratio		0.65 (0.38 - 1.01)	0.65 (0.38 - 1.17)	0.64 (0.37 - 0.83)	0.568
Neutrophils, 10^9/L		3.8 (2.6 - 4.7)	2.95 (2.3 - 4.2)	4.55 (2.9 - 5.4)	0.04
Lymphocytes, 10^9/L		1.35 (0.9 - 2.1)	1 (0.7 - 2.1)	1.55 (1 - 2.2)	0.127
Neutrophils/lymphocyts ratio		2.46 (1.43 - 4.53)	1.85 (1.35 - 7.33)	2.63 (1.77 - 3.9)	0.743
Platelets, 10^9/L		129.5 (83 - 189.5)	110 (79 - 150)	151.5 (96 - 240)	0.248
Creatinine, mg/dL		0.95 (0.78 - 1.2)	0.82 (0.71 - 1.08)	1.01 (0.83 - 1.48)	0.018
PTs/INR		1.07 (1 - 1.15)	1.11 (1.05 - 1.15)	1.03 (0.99 - 1.17)	0.137
HIV-RNA, copies/mL		377 (126 - 19621)	550 (204 - 12170)	111 (65 - 27071)	0.371
HIV-RNA, ≥50copies/mL		8 (19.5)	5 (26.3)	3 (13.6)	0.144
	Unknown	2 (4.9)	2 (10.5)	0 (0)	

Results are described by median (IQR) or frequency (%). Abbreviations: RE:recurrence; No-RE: no recurrence; ART: antiretroviral therapy; IVDU: intravenous drug users; MSM: aman who have sex with a man; FU: follow up; AFP: alpha phetoprotein; PTs/INR: prothrombin, second/international normalized ratio; AST: aspartate aminotransferase (normal values <35 IU/L); ALT: alanine aminotransferase (normal values <59 IU/L); PCHE: pseudocholinesterase; HCV: hepatitis C virus; HBsAg: hepatitis B surface antigen.
