# Supplementary material for: Survival in People Living with HIV with or without Recurrence of Hepatocellular Carcinoma after Invasive Therapy
Source: Cancers (Basel). 2023 Mar 8;15(6):1653. doi: 10.3390/cancers15061653 (PMC10046370; doi:10.3390/cancers15061653)
Supplement: Supplementary file 1 [file cancers-15-01653-s001.zip › Table S3 supplemental recurrence.pdf]

Table S3 Supplemental. Biochemistry at last evaluation of PLWH who received invasive therapy according to the outcome recurrence.

**Variable**		**Overall (number=41)**	**RE (number=19)**	**No-RE (number=22)**	**P-value**
AFP, ng/mL		13.2 (2.7 - 333.2)	35.6 (2.4 - 3066.4)	13.2 (2.9 - 31.9)	0.545
AFP, ng/mL					0.203
	<28.8	24 (61.5%)	9 (50%)	15 (71.4%)	
	≥28.8	15 (38.5%)	9 (50%)	6 (28.6%)	
AST, U/L		43 (25 - 80)	43 (18 - 79)	42 (28 - 96)	0.388
ALT, U/L		58 (32 - 158)	68 (25 - 123)	57.5 (32 - 176)	0.619
Bilirubin, mg/dL		1.05 (0.67 - 1.94)	0.87 (0.7 - 1.67)	1.51 (0.58 - 2.09)	0.228
PCHE, KU/L		5.66 (2.92 - 8.04)	4.95 (2.57 - 6.57)	5.98 (3.75 - 8.46)	0.136
CD4 cells count, mmc		343 (215 - 497)	320 (183 - 433)	374 (265 - 512)	0.245
CD8 cells count, mmc		0.7 (0.41 - 0.89)	0.77 (0.42 - 1.06)	0.66 (0.4 - 0.89)	0.657
CD4/CD8 ratio		546 (329 - 852)	449 (242 - 686)	645 (385 - 916)	0.062
Neutrophils, 10^9/L		3.9 (2.9 - 5.4)	3.3 (2.8 - 4.1)	4.1 (3.4 - 6.5)	0.137
Lymphocytes, 10^9/L		1.5 (0.9 - 2)	1.1 (0.8 - 2)	1.7 (1 - 2.2)	0.119
Neutrophils/ lymphocytes ratio		2.67 (2 - 4.2)	2.91 (2.2 - 4.2)	2.19 (1.77 - 3.9)	0.364
Platelets, 10^9/L		151 (100 - 248)	150 (100 - 264)	153 (96 - 240)	0.824
Creatinine, mg/dL		1.02 (0.83 - 1.38)	1.02 (0.81 - 1.38)	1.02 (0.83 - 1.57)	0.638
PTs/INR		1.1 (1.03 - 1.19)	1.09 (1.03 - 1.22)	1.1 (1.04 - 1.16)	0.891
HIV-RNA, copies/mL		88 (64 - 12170)	64 (62 - 12170)	11 (65 - 27071)	0.383
HIV-RNA, ≥50copies/mL		6 (14.6%)	3 (15.8%)	3 (13.6%)	0.846

Results are described by median (IQR) or frequency (%). Abbreviations: n: number; ART: antiretroviral therapy; IVDU: intravenous drug users; MSM: a man who have sex with a man; FU: follow up; AFP: alpha phetoprotein; PTs/INR: prothrombin, second/international normalized ratio; AST: aspartate aminotransferase (normal values <35 IU/L); ALT: alanine aminotransferase (normal values <59 IU/L); PCHE: pseudocholinesterase; HCV: hepatitis C virus; HBsAg: hepatitis B surface antigen.
