# Supplementary figures and images for: Survival in People Living with HIV with or without Recurrence of Hepatocellular Carcinoma after Invasive Therapy
Source: Cancers (Basel). 2023 Mar 8;15(6):1653. doi: 10.3390/cancers15061653 (PMC10046370; doi:10.3390/cancers15061653)

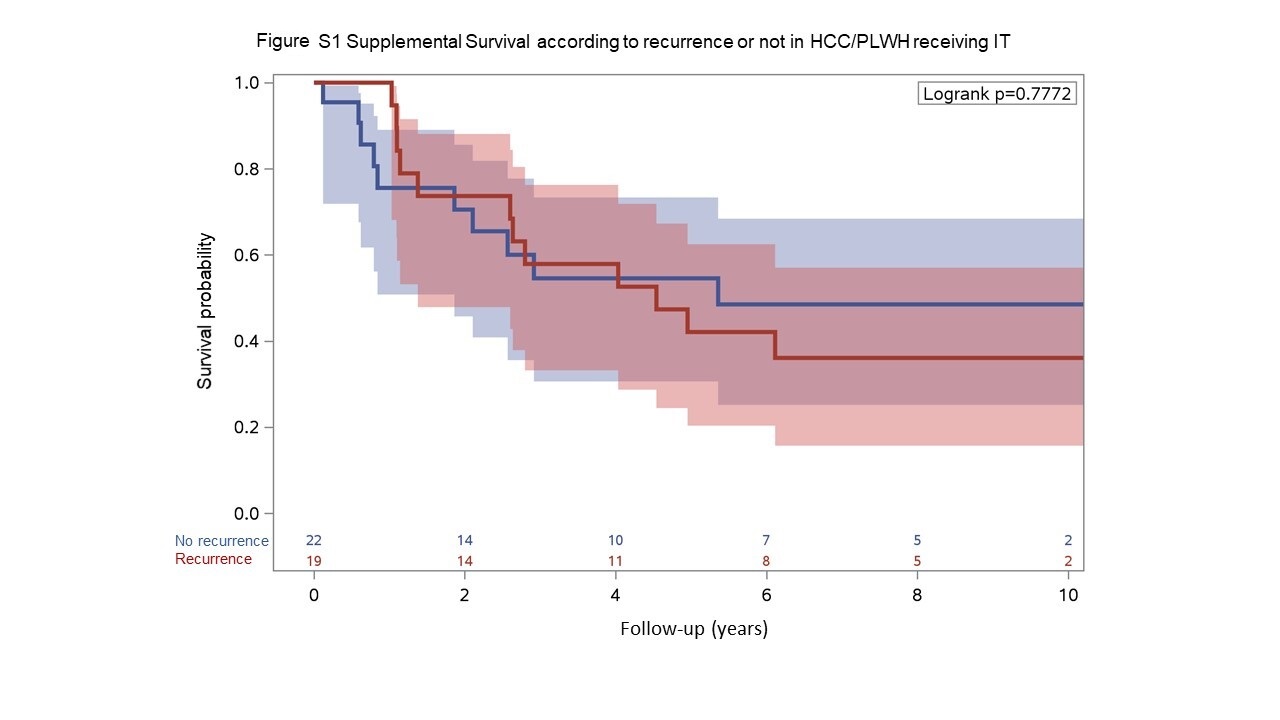

Supplement: Supplementary file 1 [file cancers-15-01653-s001.zip › figure-S1-supplementary_XKxVhLJQ.jpg]
